# Diversity of left-right symmetry breaking strategy in animals

**DOI:** 10.12688/f1000research.21670.1

**Published:** 2020-02-19

**Authors:** Hiroshi Hamada, Patrick Tam

**Affiliations:** 1 Organismal Pattterning Lab, RIKEN Center for Biosystems Dynamics Research, RIKEN, Kobe, Hyogo, Japan; 2 Embryology Unit, Children's Medical Research Institute and School of Medical Sciences, Faculty of Medicine and Health, University of Sydney, Sydney, NSW, Australia

**Keywords:** evolution, left-right asymmetry, Nodal, vertebrates

## Abstract

Left-right (L-R) asymmetry of visceral organs in animals is established during embryonic development via a stepwise process. While some steps are conserved, different strategies are employed among animals for initiating the breaking of body symmetry. In zebrafish (teleost), *Xenopus* (amphibian), and mice (mammal), symmetry breaking is elicited by directional fluid flow at the L-R organizer, which is generated by motile cilia and sensed by mechanoresponsive cells. In contrast, birds and reptiles do not rely on the cilia-driven fluid flow. Invertebrates such as *Drosophila* and snails employ another distinct mechanism, where the symmetry breaking process is underpinned by cellular chirality acquired downstream of the molecular interaction of myosin and actin. Here, we highlight the convergent entry point of actomyosin interaction and planar cell polarity to the diverse L-R symmetry breaking mechanisms among animals.

## Introduction 

The body of bilaterian animals is patterned in three axes: anterior-posterior (A-P), dorsoventral (D-V), and left-right (L-R), with L-R patterning often the last to be discerned following the breaking of bilateral symmetry. While bilaterian animals are superficially L-R symmetric, some internal organs are L-R asymmetric in terms of their shape, size, or position. Such L-R asymmetry is essential for the organs, such as the heart and the gut, to function properly. For instance, abnormal L-R asymmetry in humans and mice results in laterality defects of visceral organs often associated with severe dysfunction of the malformed heart. How L-R asymmetry is established during development has been studied in a variety of model animals ^1– 3^. The Nodal pathway acts as the left-side determinant in all vertebrates examined, as well as in some invertebrates. The molecular functionality of L-R patterning is relatively conserved, but the symmetry breaking mechanisms appear to be different among animals ^2, 4^.

## Cilia-dependent L-R symmetry breaking in the fish, frog, and mouse 

**Figure 1.**
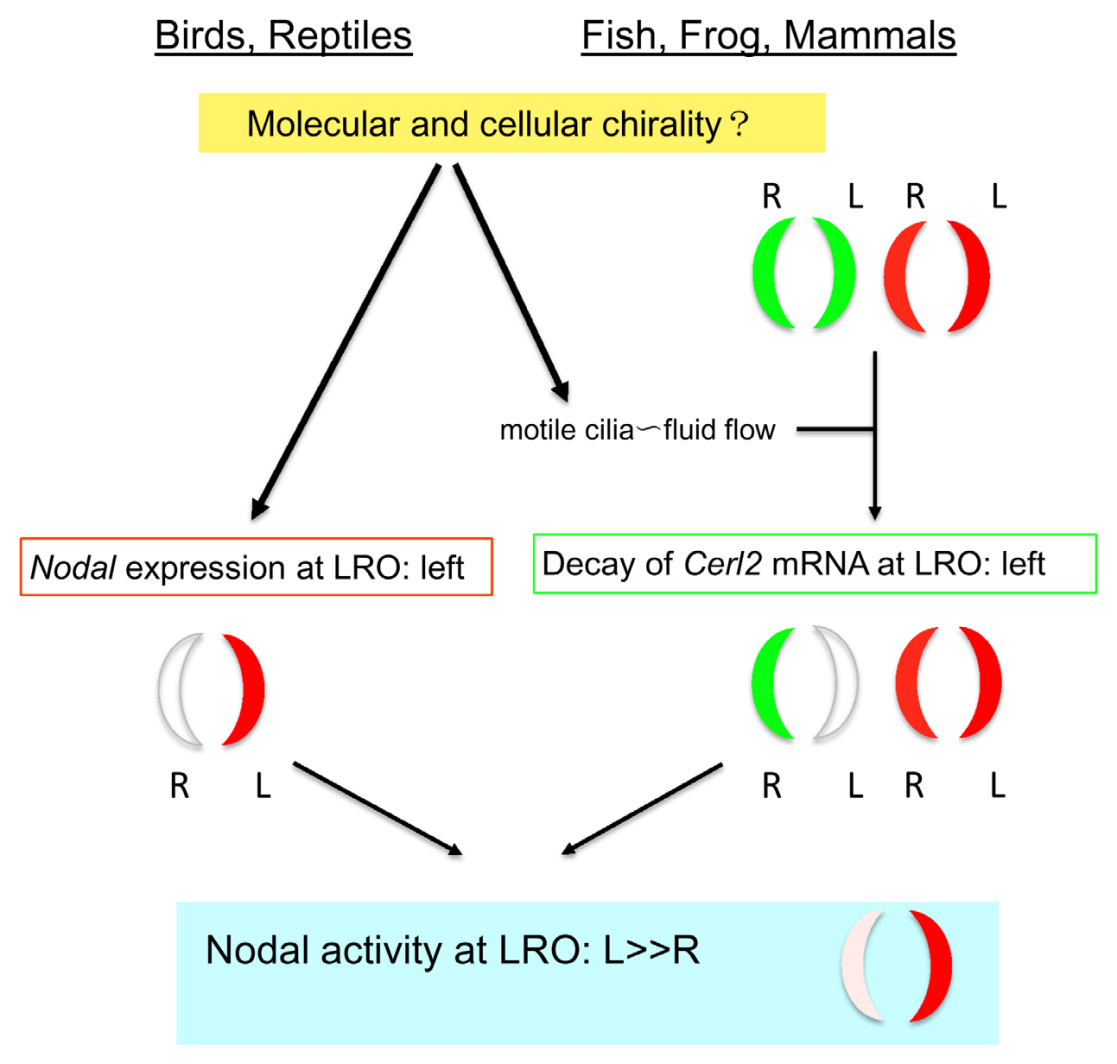
The molecular cascade of cilia dependent and non-dependent mechanisms leading to the asymmetric Nodal activity in the left-right organizer (LRO) of the vertebrate embryos. Nodal activity (red), Cerl2 activity (green). Note that both mechanisms result in asymmetric (L>R) Nodal activity at the LRO.

The L-R organizer (LRO) is an embryonic structure where L-R symmetry breaking takes place. It is located at the ventral node in the mouse, Hensen’s node in the chicken, the gastrocoel roof plate in the frog ( *Xenopus*), and Kupffer’s vesicle in zebrafish. The LRO of fish, amphibians, and mammals has motile cilia, with about 200 motile cilia in the mouse LRO, which rotate to generate the directional fluid flow across the LRO ^2, 4, 5^. The fluid flow may elicit a chemosensory ^6^ or mechanosensory ^7^ response from ciliated cells on one side of the LRO by activating the Ca ^2+^ and polycystin channels ^8– 10^, which generates a laterality cue for asymmetric tissue patterning. *Nodal* expression at the LRO is overtly bilaterally symmetric at the LRO, while the level of *Nodal* mRNA at the LRO of the mouse embryo shows subtle L-R asymmetry ^11^. This asymmetry is, however, not essential for subsequent events ^12^. In contrast, mRNA for *Cerl2/Cer2/Dand5* (encoding a Nodal antagonist) is more evidently L-R asymmetric at LRO ^13^. *Cerl2* mRNA is initially equal on both sides of the LRO, but, following the action of directional flow, *Cerl2* mRNA is repressed on the left side by an unknown mechanism that degrades the mRNA, resulting in more abundant *Cerl2* mRNA on the right side ^14, 15^. This would implicate a higher Nodal activity on the left side of the LRO ( Figure 1). This L-R asymmetric Nodal activity will be transmitted to the lateral plate mesoderm and activates the Nodal-Pitx2 cascade on the left side that confers laterality of the body plan. This molecular strategy of L-R asymmetry is common to fish, amphibians, and mammals ( Figure 1). However, LRO morphology varies substantially among mammals ^16^, and it has been suggested ^17^ that the LRO of the pig embryo does not have sufficient space for motile cilia to generate the fluid flow. While it is generally accepted that the mechanism of L-R symmetry breaking is conserved in the mammal, variations of the theme may be anticipated.

## Cilia-independent L-R symmetry breaking in birds and reptiles

Other animals deploy a mechanism of L-R symmetry breaking that is independent of motile cilia and fluid flow ^17^. In the chick, motile cilia are absent on the dorsal (luminal) side of Hensen’s node, the avian LRO. The avian *talpid2* mutant, in which the gene encoding C2CD3 that is essential for ciliogenesis is disrupted, manifests a ciliopathy phenotype (polydactyly and facial clefting), but no laterality defects ^18^. This indicates that cilia function is not required for L-R symmetry breaking in the chick. Instead, asymmetric (leftward) movement of cells around Hensen’s node accompanies L-R symmetry breaking. Such cellular rearrangement results in the asymmetric emplacement of Sonic hedgehog (Shh) and fibroblast growth factor 8 (FGF8) expressing cells, and thereby gives rise to nonequivalent signaling activity that breaks the bilateral symmetry.

Similarly, reptiles such as the Madagascar ground gecko and Chinese softshell turtle employ a cilia-independent mechanism for L-R symmetry breaking ^19^. The LRO of reptilian embryos is likely to reside at the blastopore, since the blastopore is equivalent to the Hensen’s node in birds ^20, 21^. Interestingly, *Cerl2*, a target gene of the fluid flow in cilia-dependent vertebrates, is absent in the genome of reptiles and birds, suggesting that the *Cerl2* gene may have been lost during evolution. In the cilia-independent vertebrates, *Nodal* expression at the LRO is inherently asymmetric (L>>R), rendering higher Nodal activity at the left side of the LRO ( Figure 1) ^22^, which may have eliminated the requisite function of the fluid flow driven by motile cilia. Therefore, the cilia-dependent and -independent vertebrates employ different strategies to achieve a common outcome: L-R asymmetric (left-sided) Nodal activity at the LRO ( Figure 1).

However, there are differences between chick and reptile embryos. Unlike in chick embryos, the expression of *Shh* and *Fgf8* in reptile embryos was bilaterally comparable initially ^22^. It would be imperative to understand the mechanism that leads subsequently to L-R asymmetric *Nodal* expression at the reptilian LRO.

## Distinct mechanisms in invertebrates: *Drosophila* and snails

**Figure 2.**
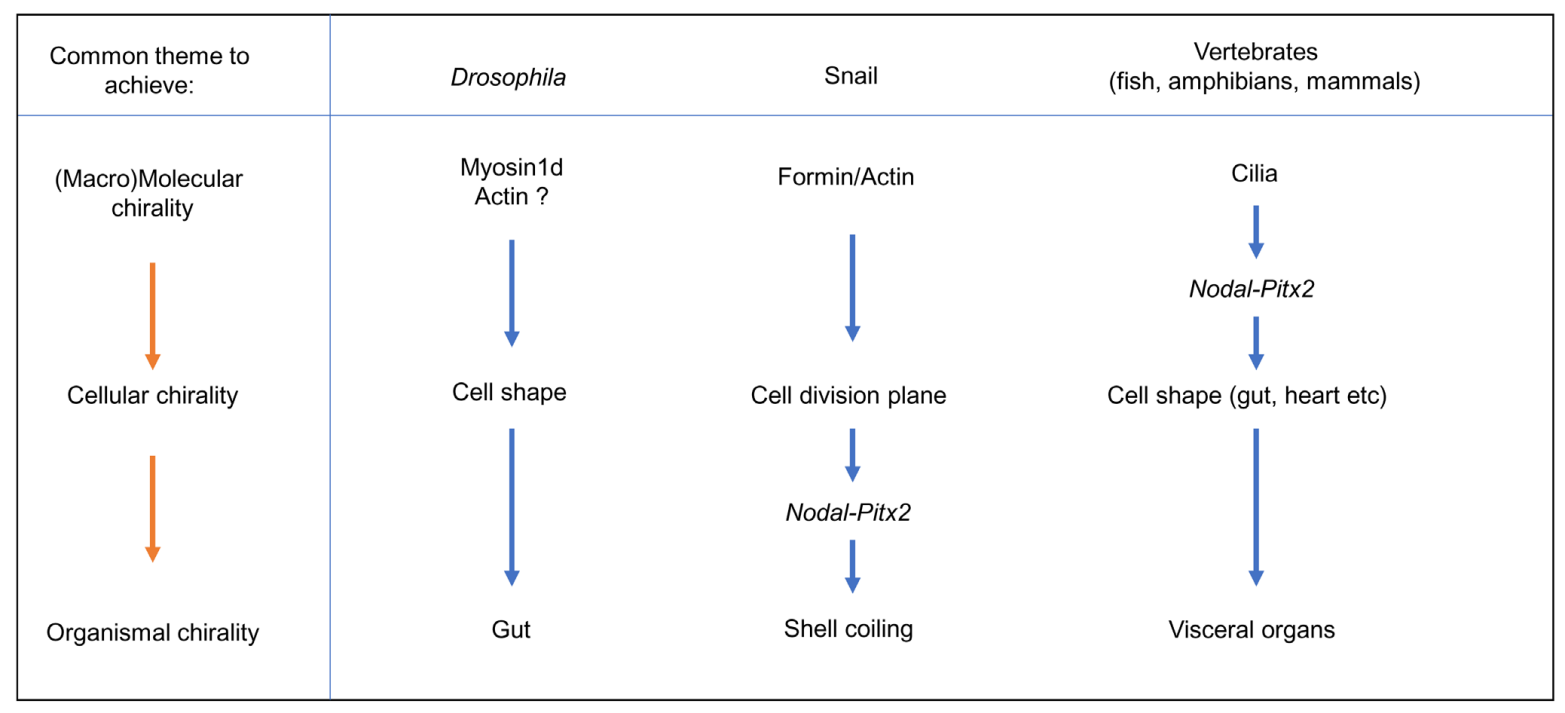
Distinct mechanism of the specification of left-right asymmetry of organs and whole organism in *Drosophila*, snails, and vertebrates. Note that different animal species use variations of the common theme (in the left-hand box) to establish L-R asymmetry. See the text for details.

Snails are spiralians that display directional coiling of the shell, a vivid example of L-R asymmetry in animals ^23^. As in other organisms, this asymmetry (chirality) is regulated by left-sided expression of *Nodal* and *Pitx2* at embryonic stages ^24^. However, the event that determines the direction of shell coiling takes place at a very early stage ( Figure 2). Snails undergo a unique spiral cleavage at the third to fifth cell divisions, and the handedness of the spiral cleavage at this early stage determines the direction of shell coiling at a later stage ^25, 26^. At the third cell division (from the four- to eight-cell stage), embryos with a quartet of micromeres that rotates in a clockwise direction relative to their sister macromeres will develop into dextral individuals. In contrast, those with micromeres rotating in an anticlockwise direction become sinistral embryos. Mechanical manipulation of the third-cleavage chirality (for example, by continuous pushing of the first quartet of micromeres being generated in the direction opposite to the normal direction with glass rods) can reverse not only the left-sided *Nodal* expression in the manipulated embryo but also the direction of shell coiling ^27^, suggesting that, in the snail embryo, micromere chirality drives the asymmetry context of Nodal activity.

Genetics has shown that L-R asymmetry in snails may be determined by a single gene (or a single locus) that functions maternally ^28, 29^. The genus *Lymnaea* is dimorphic, with both dextral (the dominant type) and sinistral (the recessive type) individuals existing within a given species. The identity of this L-R determining gene is not known, but it might be expected to regulate cytoskeletal dynamics at early development. Of note in this regard is that Formin, a Diaphanous-related protein that associates with filament tips and mediates the elongation of actin filaments, can impact on the direction of shell coiling in the pond snail ^30^ and in the freshwater snail *Lymnaea stagnalis* ^31^ ( Figure 2). *Formin* mRNA is asymmetrically distributed to one macromere at the two-cell and four-cell stages ^31^. These observations suggest that Formin may be the chiral molecule responsible for L-R symmetry breaking in snails.

L-R asymmetry in *Drosophila* is manifested by the rotation of male genitalia and looping of the larval and adult gut. In *Drosophila* males, the genital plate undergoes a 360-degree clockwise (when viewed from the posterior side) rotation during the pupal stage ^32^. This clockwise (dextral) direction is preserved among the Drosophilidae, while no sinistral species is known so far. The embryonic hindgut in *Drosophila* is formed initially as a bilaterally symmetric structure, but it later undergoes a 90-degree anticlockwise (when viewed from the posterior side) rotation that subsequently results in dextral looping. The adult gut, which develops from larval primordia, also shows directional looping. It may be noted that a similar pattern of rotation of the epithelium lining the anterior intestinal portal that heralds the directionality of rotation of the foregut and the adjacent heart tube is found in the mouse embryo ^33^.

Genetic screening of mutants with altered L-R asymmetry has identified the *Myo31DF* gene as a general L-R determinant in *Drosophila* ^34, 35^ ( Figure 2). The direction of rotation of the male genitalia and the embryonic gut as well as the looping of the adult hindgut were all reversed in the *Myo31DF* mutant. *Myo31DF* encodes a type ID unconventional myosin (Myo31DF, also known as MyoID), an actin-based motor protein that is expressed in the gut epithelium. Both calmodulin binding and ATP-binding motifs of the Myo31DF protein appear to be essential for its function in L-R asymmetric organ development. Myo31DF binds β-catenin and the atypical cadherin Dachsous ^36^ and is associated with DE-cadherin ( *Drosophila* E-cadherin) via β-catenin ^37^. The interaction of Myo31DF with the intracellular domain of Dachsous is required for embryonic gut looping ^36^. The Myo31DF–Dachsous interaction may promote the transfer of L-R information to neighboring precursor cells of the hindgut.

Hindgut epithelial cells manifest L-R asymmetry ^38^ even before the embryonic hindgut begins its directional rotation, with the cell boundary surfaces showing more leftward-tilt than rightward-tilt at the cell boundaries. Furthermore, the centrosome is preferentially located in the right-posterior region of hindgut epithelial cells, and DE-cadherin is more abundant along the leftward-tilted cell boundaries. Such asymmetry (planar cell shape chirality) disappears after the gut rotation is complete, and it is reversed in the *Myo31DF* mutant, suggesting that this intrinsic cell chirality is responsible for L-R asymmetric morphogenesis. In a similar context, epithelial cells of the male genitalia exhibit chirality before directional rotation ^39^, with more rightward-tilted cell boundaries and a higher distribution of myosin II along the rightward boundaries. A recent study implied that the planar cell shape chirality may lead to cell sliding, whereby epithelial cells change their position relative to their neighbors by directional displacement while maintaining cell–cell contact, during the rotation of the embryonic hindgut ^40^. The asymmetric cell sliding converts the global pattern of cell chirality into directional twisting of the epithelial tube and possibly the rotation of the male genitalia. Misexpression of Myo1d in *Drosophila* reversed the directional twisting of cells, organs, and the whole body, suggesting that Myo1d may be instrumental for generating chiral morphology, at least in *Drosophila* ^41^.

Of interest, myosin 1d, the ortholog of *Drosophila* Myo31DF, is also required for laterality in *Xenopus* ^42^ and zebrafish ^43, 44^. Myosin 1d in the frog and zebrafish appears to act through the Planar Cell Polarity (PCP) pathway. Myosin 1d in zebrafish appears to regulate vacuolar trafficking in epithelial cells of Kupffer’s vesicle and is required for the formation of this structure with a proper size and spherical lumen ^44^. These new findings suggest that the unconventional myosin ortholog acts as a *driver of L-R asymmetry* common to the invertebrates and vertebrates with a ciliated LRO. An exception is found in rats lacking myosin 1d, which manifest PCP defects in multi-ciliated airway epithelial cells but body laterality remains normal ^45^. The role of the myosin orthologs in L-R asymmetry thus appears to be largely, but not universally, conserved between arthropods and chordates.

## Does molecular and cellular chirality underpin L-R asymmetry? 

Chirality is manifested in individual cells, even those in culture. Human umbilical vein endothelial cells, human vascular mesenchymal cells, and mouse C2C12 myoblasts were found to generate a chiral pattern when plated on a micropatterned surface ^46^. The pattern of chirality was cell line dependent, with some showing a clockwise and others an anticlockwise alignment. Of note, the chirality manifested by C2C12 cells was resistant to the microtubule-disrupting agent nocodazole but was abolished by the microfilament-disrupting agents latrunculin A and cytochalasin D, suggesting that cell chirality depends on actomyosin function but not on microtubules. Cultured cells also show chirality in their motion, with melanophores from zebrafish ^47^ and fibroblasts from human foreskin ^48^ manifesting chiral swirling. Such unidirectional rotational movement appeared to depend on the actin cytoskeleton, in particular on Formin-mediated polymerization of actin, but not influenced by microtubules. Immobilized Formin has been shown to mediate the rotation of helical actin filaments in a clockwise direction relative to itself ^49^, and this clockwise rotation may lead to a rightward tilting of actin fibers. It is tempting to speculate that such intracellular chirality elicits L-R asymmetry of organs. Of interest, cardiac cells in the developing chick embryo also show intrinsic chirality and a rightward polarization of the Golgi complex ^50^. The intracellular chirality may also underpin L-R asymmetry of the whole organism, which may indeed be the case at least in some animals such as *Drosophila* and snails.

## Outstanding issues 

We have now gleaned a better understanding of the construction and the putative mode of action of the LRO of vertebrate embryos for the specification of L-R asymmetry of organs and the body. From the vantage point of recent knowledge of L-R asymmetry of the invertebrates, several pressing issues would demand further clarification. They include the following: (i) what is the precise function of myosin 1d in *Drosophila*? How does it induce cellular chirality? (ii) How does Formin-regulated symmetry breaking lead to asymmetric expression of *Nodal* in snails? (iii) Are myosin 1d and an actin regulator, such as Formin, involved in L-R symmetry breaking in amniotes? If they are involved, what is their precise role, and is this the origin of L-R asymmetry? (iv) How do non-mammalian amniotes (reptiles and birds) break L-R symmetry without motile cilia and directional fluid flow?
